# The Autocrine FGF/FGFR System in both Skin and Uveal Melanoma: FGF Trapping as a Possible Therapeutic Approach

**DOI:** 10.3390/cancers11091305

**Published:** 2019-09-04

**Authors:** Sara Rezzola, Roberto Ronca, Alessandra Loda, Mohd Imtiaz Nawaz, Chiara Tobia, Giuseppe Paganini, Federica Maccarinelli, Arianna Giacomini, Francesco Semeraro, Marco Mor, Marco Presta

**Affiliations:** 1 Department of Molecular and Translational Medicine, University of Brescia, 25123 Brescia, Italy; 2 Eye Clinic, Department of Medical and Surgical Specialties, Radiological Sciences and Public Health, University of Brescia, 25123 Brescia, Italy; 3 Department of Food and Drug, University of Parma, 43125 Parma, Italy

**Keywords:** FGF, FGF receptors, liver metastasis, tumor grafts, uveal melanoma, zebrafish

## Abstract

Fibroblast growth factors (FGFs) play non-redundant autocrine/paracrine functions in various human cancers. The Cancer Genome Atlas (TCGA) data mining indicates that high levels of FGF and/or FGF receptor (FGFR) expression are associated with reduced overall survival, chromosome 3 monosomy and *BAP1* mutation in human uveal melanoma (UM), pointing to the FGF/FGFR system as a target for UM treatment. Here, we investigated the impact of different FGF trapping approaches on the tumorigenic and liver metastatic activity of liver metastasis-derived murine melanoma B16-LS9 cells that, similar to human UM, are characterized by a distinctive hepatic tropism. In vitro and in vivo experiments demonstrated that the overexpression of the natural FGF trap inhibitor long-pentraxin 3 (PTX3) inhibits the oncogenic activity of B16-LS9 cells. In addition, B16-LS9 cells showed a reduced tumor growth and liver metastatic activity when grafted in PTX3-overexpressing transgenic mice. The efficacy of the FGF trapping approach was confirmed by the capacity of the PTX3-derived pan-FGF trap small molecule NSC12 to inhibit B16-LS9 cell growth in vitro, in a zebrafish embryo orthotopic tumor model and in an experimental model of liver metastasis. Possible translational implications for these observations were provided by the capacity of NSC12 to inhibit FGF signaling and cell proliferation in human UM Mel285, Mel270, 92.1, and OMM2.3 cells. In addition, NSC12 caused caspase-3 activation and PARP cleavage followed by apoptotic cell death as well as β-catenin degradation and inhibition of UM cell migration. Together, our findings indicate that FGF trapping may represent a novel therapeutic strategy in UM.

## 1. Introduction

Uveal melanoma (UM) is the most common primary intraocular malignancy in adults. Its occurrence increases with age and its incidence is more than 20 per million per year [1,2]. Current treatments of primary UM include enucleation, radiotherapy, transpupillary thermotherapy, and local resection, achieving a control of local tumor in up to 97% of treated cases. Anyway, the mortality rate is high because of the frequent occurrence of metastases by hematogenous dissemination: almost 50% of all UM patients develop metastatic disease, mainly in the liver, with a current 5-year mortality ranging from 26% to 32% [1,2,3]. In the last years, a better understanding of UM biology has provided new indications for the development of efficacious adjuvant therapies and for the treatment of the metastatic disease [4]. At present, numerous chemotherapy-, targeted therapy-, immunotherapy-, and liver directed therapy-based clinical trials are in progress [4,5,6]. However, as of today, no specific systemic treatment has been approved, indicating that novel biologically-based therapies are urgently required.

The fibroblast growth factor (FGF)/FGF receptor (FGFR) system plays a pivotal role in different tumor types, leading to autocrine/paracrine stimulation of tumor cell proliferation and angiogenesis [7,8,9]. The evidence that UM cell cultures express and secrete large quantities of FGFs suggests that an FGF/FGFR autocrine loop of stimulation exists also in these cells [10,11,12], implying the FGF/FGFR system in UM progression [13] and pointing to this pathway as a possible alternative therapeutic target in UM.

The soluble pattern recognition receptor long pentraxin-3 (PTX3) is a member of the pentraxin family produced locally in response to inflammatory signals [14,15]. Previous observations have shown that PTX3 binds various FGFs, including FGF2, FGF6, FGF8b, FGF10, and FGF17, and inhibits FGF-dependent angiogenic responses [16,17,18]. Accordingly, transgenic PTX3 overexpression impairs efficaciously the activation and signaling of the FGF/FGFR system in FGF-driven tumors, thus affecting tumor growth and metastasis [16,17,19].

Recently, the PTX3-derived small molecule NSC12 has been identified as the first orally active pan-FGF trap able to inhibit FGFR activation and tumor growth in various FGF-dependent murine and human tumor models [20,21]. Notably, extracellular FGF traps, including NSC12, appear to be devoid of the toxicities associated with tyrosine kinase FGFR inhibitors [21,22], thus representing a potential alternative option to inhibit the FGF/FGFR system in cancer. On this basis, in this work we investigated the impact of a PTX3/NSC12-based, FGF trapping approach in the murine B16-LS9 melanoma model and on human UM cell lines.

## 2. Results

### 2.1. The FGF/FGFR System Is Upregulated in Human Uveal Melanoma

**Figure 1:**
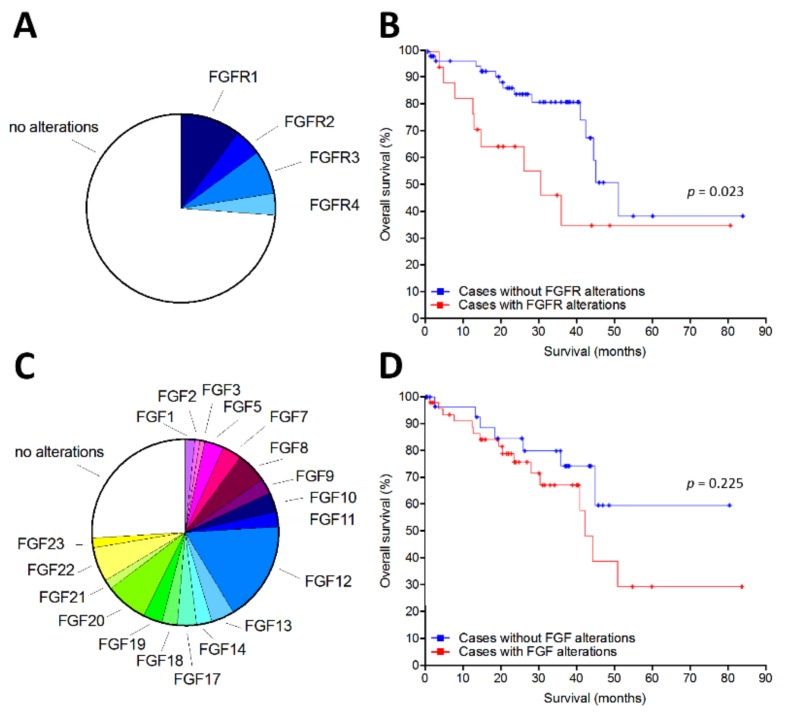
Fibroblast growth factor receptor (FGFR) and fibroblast growth factor (FGF) overexpression in human primary uveal melanoma (UM). Analysis of The Cancer Genome Atlas (TCGA) dataset was performed on a cohort of 80 UM patients. (**A**) Pie chart showing the percentage of samples with mRNA overexpression of the different FGFRs. (**B**) Overall survival of patients with or without FGFR alterations. (**C**) Pie chart showing the percentage of samples with mRNA overexpression of different members of the FGF family. Some samples showed the overexpression of more than one FGF family member. (**D**) Overall survival of patients with or without FGF alterations.

Data mining was performed on The Cancer Genome Atlas (TCGA) UM PanCancer Atlas dataset (http://www.cbioportal.org/study?id=uvm_tcga_pan_can_atlas_2018) to investigate the expression of all the members of the FGFR and FGF families in a UM cohort of 80 patients. We found that FGFRs are overexpressed in 21% of UM cases, these alterations being associated to a poor prognosis when compared to UM cases without FGFR alterations (*p* = 0.023, log-rank test; median survival equal to 31 and 52 months for cases with or without FGFR alterations, respectively) (Figure 1A,B). Moreover, overexpression of one or more FGFs was detected in 61% of UM patients (Figure 1C). Again, FGF overexpression appears to be associated to a reduced survival in UM patients, even though the difference between the two groups did not reach the statistical significance (Figure 1D).

**Figure 2:**
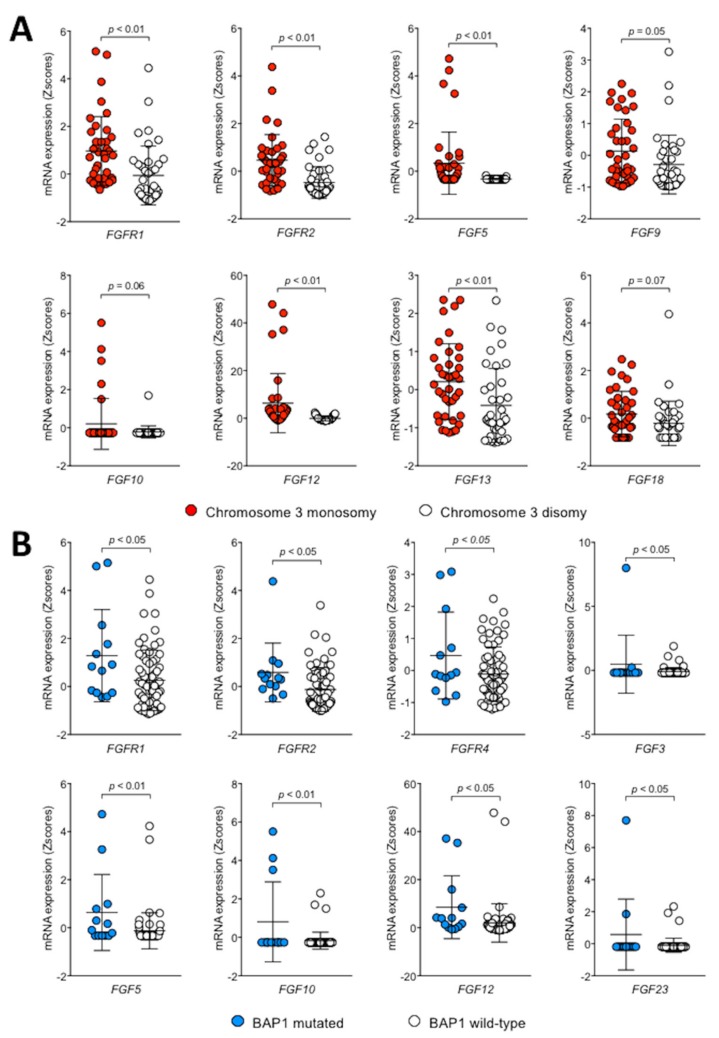
Correlation between FGF/FGFR expression and chromosome 3 /*BAP1* status in UM. Analysis of the expression of all members of the FGFR and FGF families was performed on the cohort of 80 UM patients present in the UM TCGA dataset. FGF/FGFR genes that showed a significant differential expression between chromosome 3 (monosomic, red symbols; disomic, open symbols) and *BAP1* (mutated, blue symbols; wild-type, open symbols) status.

In the recent years, analysis of the genetic alterations has identified subsets of UM patients with distinct molecular signatures [23]. Among them, UMs with loss of chromosome 3 are characterized by a poor prognosis when compared to chromosome 3 disomic lesions. On this basis, we performed a preliminary analysis of FGF/FGFR expression in chromosome 3 monosomic and disomic UMs of the cohort of 80 patients present in the TCGA dataset. The results demonstrate that high-risk chromosome 3 monosomic tumors are characterized by a higher expression of FGFR1 and FGFR2, as well as of FGF5, FGF9, FGF10, FGF12, FGF13, and FGF18 (Figure 2).

The tumor suppressor BAP1 plays a key role in UM progression and monosomy of chromosome 3 is highly associated with the loss of nuclear expression of BAP1, frequently related to loss-of-function *BAP1* mutations (see [24] and references therein). Accordingly, 13 out of the 40 chromosome 3 monosomic tumors present in the UM TCGA dataset carried a *BAP1* mutation, absent in the 40 disomic specimens. Notably, various members of the FGF/FGFR families appear to be upregulated in this subset of *BAP1* mutated tumors when compared to *BAP1* wild-type UMs (Figure 2).

Together, these data point to a potential role of the FGF/FGFR axis in UM.

### 2.2. PTX3 Inhibits the Tumorigenic and Metastatic Activity of Murine B16-LS9 Cells

B16-LS9 cells is a murine cell line originated from a B16-F1 liver metastasis and characterized by a unique tropism for the hepatic tissue [25]. Even though of cutaneous origin, this cell line has been utilized as an experimental model to investigate the mechanisms responsible for UM liver tropism [26,27,28] and drug evaluation for UM treatment [29,30,31].

**Figure 3:**
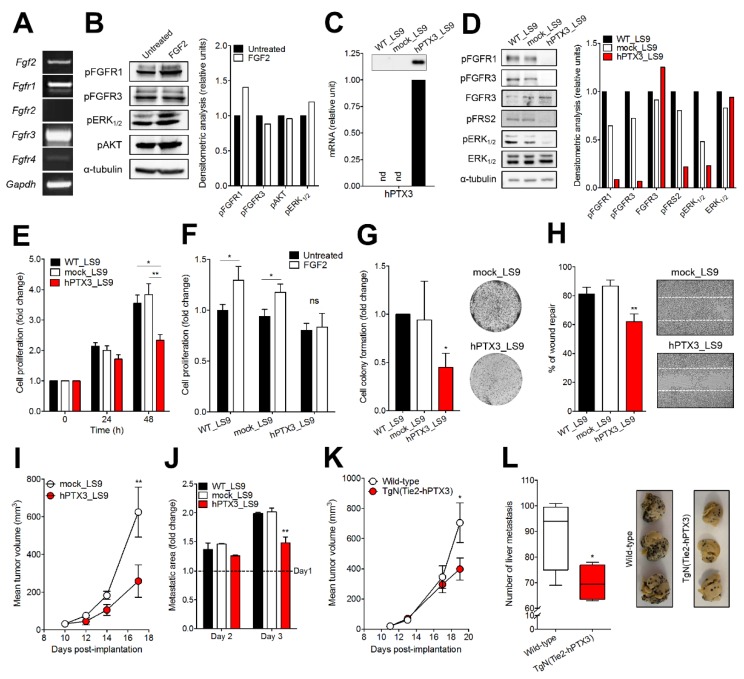
Effect of long-pentraxin 3 (PTX3) overexpression on B16-LS9 cells. (**A**) RT-PCR analysis of *Fgf2* and *Fgfr* expression in B16-LS9 cells. (**B**) Western blot analysis of the phosphorylation of FGFR1 and FGFR3 and of the downstream signaling proteins ERK_1/2_ and AKT in B16-LS9 cells following 30 min treatment with 30 ng/mL FGF2. (**C**) RT-PCR analysis of human PTX3 (hPTX3) expression in WT_LS9, mock_LS9, and hPTX3_LS9 cells. Inset: Western blot analysis of PTX3 protein levels in the extracts of the same cells. (**D**) Western blot analysis of the phosphorylation of FGFR1, FGFR3, FRS2, and ERK_1/2_ proteins in WT_LS9, mock_LS9, and hPTX3_LS9 cell extracts. (**E**) WT_LS9, mock_LS9, and hPTX3_LS9 cells were seeded in 48-well plates at 10^4^ cells/well in medium containing 0.4% FBS. After 24 h (T_0_), medium was changed, and cell were counted 24 and 48 h thereafter. Data are the mean ± SEM of three independent experiments in triplicate. (**F**) Cells were seeded as in (**E**). At T_0_, cells were treated with 30 ng/mL FGF2 and counted 24 h thereafter. Data are the mean ± SEM of three independent experiments in triplicate (**G**) WT_L69, mock_LS9, and hPTX3_LS9 cells were seeded at 50 cells/cm^2^. After 10 days, cell colonies were stained with crystal violet and quantified by computerized image analysis. Representative images of mock_LS9 and hPTX3_LS9 cell colonies are shown on the right. Data are the mean ± SEM of 15 fields for each triplicate sample. (**H**) A mechanical wound was performed in WT_LS9, mock_LS9, and hPTX3_LS9 cell monolayers. After 18 h, cell migration at the leading edge of the wound was quantified by computerized image analysis. Representative images of wounded mock_LS9 and hPTX3_LS9 cell monolayers are shown on the right. Data are the mean ± SEM of six microscopic fields. (**I**) Mock_LS9 and hPTX3_LS9 cells were injected subcutaneously (s.c.) in syngeneic mice at 50,000 cells/graft and tumor growth was measured with calipers. Data are the mean ± SEM (*n* = 16). (**J**) Red fluorescent WT_LS9, mock_LS9, and hPTX3_LS9 cells were injected into the bloodstream of 48 hours post fertilization (hpf) zebrafish embryos (80–100 cells/embryo). During the next 3 days, the growth of fluorescent metastases in the tail vascular plexus was quantified by fluorescence microscopy followed by computerized image analysis. Data are the mean ± SEM of three independent experiments (*n* = 20) and were normalized to metastasis areas at day 1. (**K**) WT_LS9 cells were injected s.c. in wild-type and transgenic TgN (Tie2-hPTX3) mice (50,000 cells/graft) and tumor growth was measured with calipers. Data are the mean ± SEM (*n* = 18). (**L**) WT_LS9 cells were injected into the spleen of wild-type and transgenic TgN (Tie2-hPTX3) mice (20,000 cells/graft). After 14 days, livers were harvested, and metastases were counted. Representative images of harvested livers are shown on the right. Data are the mean ± SEM (*n* = 5). In (B) and (D), the right panel shows the densitometric analysis of immunoreactive bands normalized to α-tubulin protein levels. *p < 0.05; **p < 0.01, Student’s *t*-test (F,L), one-way (E,H,J) and two-way (I,K) analysis of variance.

As shown in Figure 3A, B16-LS9 cells express FGF2 and its receptors FGFR1 and FGFR3. The autocrine production of FGF2, and possibly of other FGF family members, leads to a basal activation of FGFRs and of the downstream signaling proteins ERK_1,2_ and AKT (Figure 3B). Addition of exogenous FGF2 to B16-LS9 cells causes no or only a very modest further increase in FGFR phosphorylation and of the downstream signaling mediators phospho-AKT and phospho-ERK_1,2_, thus confirming the presence of a constitutive autocrine FGF/FGFR loop of activation in these cells under basal cell culture conditions [12].

To assess the capacity of the natural FGF trap PTX3 to suppress the constitutive activation of the FGF/FGFR system, B16-LS9 cells were transfected with a pBABE/Puro vector harboring the full-length human PTX3 (hPTX3) cDNA or with an empty vector. Stable hPTX3_LS9 and mock_LS9 cell populations were generated by puromycin selection. After selection, transfectants were assessed for hPTX3 expression and secretion by RT-PCR and Western blotting, respectively (Figure 3C). The production of hPTX3 leads to a significant inhibition of FGFR1, FGFR3, FRS2, and ERK_1,2_ activation (Figure 3D) followed by a reduction of the proliferative capacity of hPTX3_LS9 cells in respect to control non-transfected (WT_LS9) and mock_LS9 cells (Figure 3E). Accordingly, hPTX3_LS9 cells were unable to proliferate in response to exogenous FGF2 stimulation (Figure 3F). In addition, the capacity of hPTX3_LS9 cells to form colonies when seeded at low cell density and to repair a wounded cell monolayer was significantly reduced in respect to control cells (Figure 3G,H).

To assess the impact of PTX3 overexpression on the tumorigenic activity of B16-LS9 cells, hPTX3_LS9 and mock_LS9 cells were injected subcutaneously (s.c.) in syngeneic mice. As shown in Figure 3I, hPTX3_LS9 grafts show a reduced rate of growth when compared to mock tumors. Next, to assess the capacity of PTX3 to affect the metastatic activity of B16-LS9 cells, WT_LS9, mock_LS9 and hPTX3_LS9 cells were injected into the blood circulation of zebrafish embryos at 48 hours post fertilization (hpf) and the growth of micrometastases in the tail vascular plexus was followed [32]. As shown in Figure 3J, PTX3 overexpression results in a significant reduction of the size of micrometastases evaluated 3 days after cell injection.

These observations prompted us to investigate whether also the systemic/stromal overexpression of PTX3 may exert a significant impact on the tumorigenic and metastatic activity of B16-LS9 cells. To this purpose, WT_LS9 were grafted in syngeneic transgenic TgN (Tie2-hPTX3) mice in which PTX3 expression is driven by the endothelial specific promoter Tie2. These animals are characterized by high levels of PTX3 protein in the bloodstream and by its accumulation in the stroma of different organs [21]. As shown in Figure 3K, stroma accumulation of PTX3 reduced the growth of B16-LS9 tumors grafted s.c. in transgenic mice when compared to wild-type animals. In addition, a significant difference in liver colonization was observed between wild-type and TgN (Tie2-hPTX3) animals following intrasplenic injection of B16-LS9 cells (Figure 3L). Together, our data indicate that the PTX3 inhibits the tumorigenic and metastatic activity of B16-LS9 cells.

### 2.3. The Pan-FGF Trap NSC12 Inhibits the Tumorigenic and Metastatic Activity of Murine B16-LS9 Cells

PTX3 is a 340 kDa protein composed of eight protomers, with a complex proteinaceous structure that hampers its pharmacological exploitation. To overcome these limitations, NMR data and pharmacophore modeling of PTX3/FGF2 interaction were used in our laboratory to identify the PTX3-derived small molecule NSC12 as the first orally active pan-FGF trap able to inhibit FGFR activation and tumor growth in various FGF-dependent murine and human tumor models [20,21].

**Figure 4:**
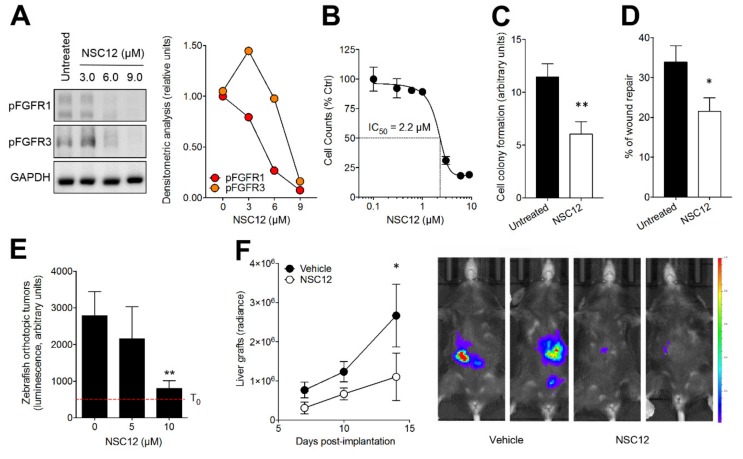
Effect of the pan FGF-trap NSC12 on B16-LS9 cells. (**A**) Western blot analysis of FGFR1 and FGFR3 phosphorylation in B16-LS9 cells treated for 12 h with increasing concentrations of NSC12. The right panel shows the densitometric analysis of immunoreactive bands normalized to GAPDH protein levels. (**B**) Effect of NSC12 treatment on the proliferation of B16-LS9 cells. Viable cells were counted after 24 h of incubation with increasing concentrations of NSC12. Data are the mean ± SEM (*n* = 3). (**C**) B16-LS9 cells were seeded at 50 cells/cm^2^ and treated with 2.5 µM NSC12. After 10 days, cell colonies were stained with crystal violet and quantified by computerized image analysis. Data are the mean ± SEM of 15 fields for each triplicate sample. (**D**) A mechanical wound was performed in a B16-LS9 cell monolayer followed by incubation with 3.0 µM NSC12. After 18 h, cell migration at the leading edge of the wound was quantified by computerized image analysis. Data are the mean ± SEM of six microscopic fields. (**E**) B16-LS9-luc cells were injected into the eye of 48 hpf zebrafish embryos (100 cells/embryo). Then, embryos were incubated with increasing concentrations of NSC12 at T_0_. Tumor growth was evaluated 3 days after grafting by measuring the cell luminescence signal. Data are the mean ± SEM (*n* = 20). (**F**) B16-LS9-*luc* cells were grafted in the liver of syngeneic mice (50,000 cells/graft). Next, vehicle or NSC12 (7.5 mg/kg) were injected i.p. every other day and tumor growth was imaged with IVIS Lumina III for the following 14 days. Data are the mean ± SEM (*n* = 9). Representative images of control and NSC-12 treated mice imaged 14 days after grafting are shown on the right. *p < 0.05; **p < 0.01, Student’s *t*-test (**C,D**), one-way (**E**) and two-way (**F**) analysis of variance.

As shown in Figure 4A, treatment of B16-LS9 cells with increasing concentrations of NSC12 inhibits FGFR phosphorylation, thus affecting autocrine, FGF-mediated cell proliferation (IC_50_ = 2.2 µM, Figure 4B). Accordingly, NSC12 treatment hampered the capacity of B16-LS9 cells to form colonies when seeded at low density and to repair a wounded cell monolayer (Figure 4C,D).

To evaluate the effect of NSC12 on the oncogenic potential of B16-LS9 cells, we implemented a novel orthotopic xenograft assay in which luciferase-transfected cells (B16-LS9-luc cells) were injected into the eye of zebrafish embryos at 48 hpf. Then, embryos were transferred in fish water in the absence or in the presence of 5.0 or 10 µM NSC12. NSC12 treatment resulted in a significant inhibition of the growth of grafted cells when assessed 3 days after injection (Figure 4E). Accordingly, administration of NSC12 (7.5 mg/kg i.p. every other day) hampered the growth of B16-LS9-luc cells grafted into the liver of wild-type mice (Figure 4F).

### 2.4. FGF Trapping Inhibits Human FGF/FGFR Signaling and Proliferation in UM Cells

The capacity of the pan-FGF trap NSC12 to inhibit FGF/FGFR signaling in human UM was investigated on three cell lines originating from human primary UM lesions (Mel285, Mel270, and 92.1 cells) and one cell line originating from a human UM metastasis (OMM2.3 cells). The major molecular alterations of these cell lines are summarized in Table 1 (see [33] and references therein).

**Figure 5:**
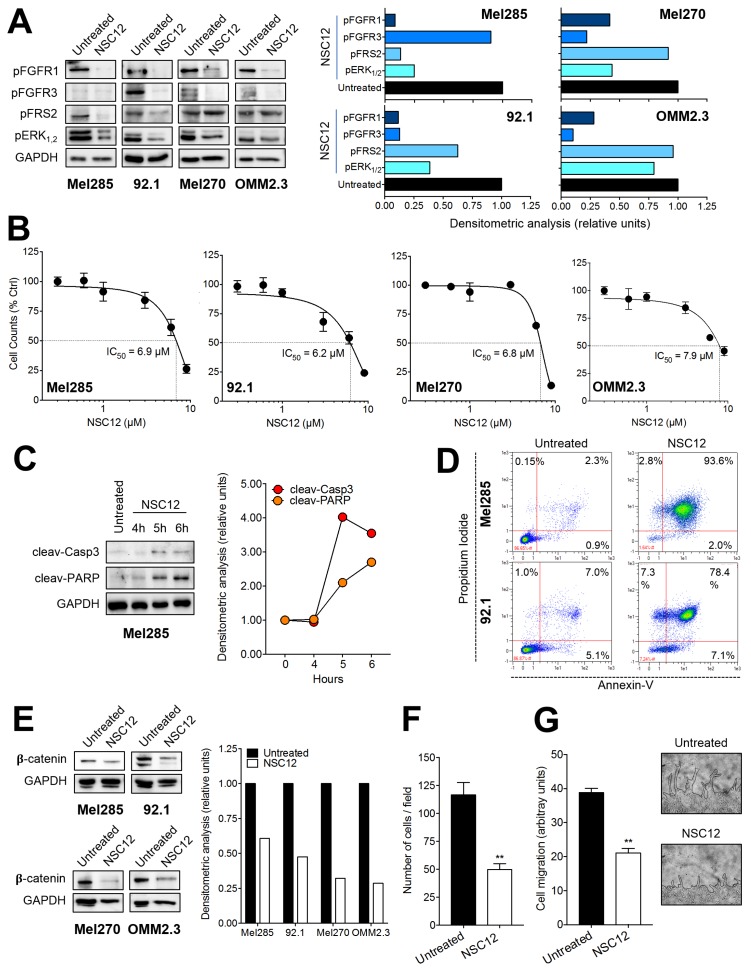
Effect of the pan FGF-trap NSC12 on human UM cells. (**A**) Western blot analysis of the phosphorylation of FGFR1 and FGFR3 and of the downstream signaling proteins FRS2 and ERK_1/2_ in Mel285, 92.1, Mel270, and OMM2.3 cells after 3 h treatment with 15 µM NSC12. (**B**) Effect of NSC12 treatment on the proliferation of UM cells. Viable cells were counted after 24 h of incubation with increasing concentrations of NSC12. Data are the mean ± SEM (*n* = 3). (**C**) Kinetics of PARP and caspase-3 cleavage following incubation of MEL285 cells with 15 µM NSC12. (**D**) Cytofluorimetric analysis of apoptosis induced in Mel285 cells (upper panels) and 92.1 cells (lower panels) after 12 h treatment with 15 µM NSC12. (**E**) Western blot analysis of the levels of β-catenin in Mel285, 92.1, Mel270, and OMM2.3 cells after 3 h treatment with 15 µM NSC12. (**F**) Boyden chamber chemotaxis assay performed on Mel285 cells treated for 4 h with 6.0 µM NSC12. Data are the mean ± SEM of five fields for each triplicate sample. (**G**) A mechanical wound was performed in a Mel285 cell monolayer followed by 18 h incubation with 6.0 µM NSC12. After 18 h, cell migration at the leading edge of the wound was quantified by computerized image analysis. Representative images of untreated and NSC12-treated cells are shown on the right (black lines highlight the front of cell migration). Data are the mean ± SEM of 6 microscopic fields. In (**A,C,D**) the right panel shows the densitometric analysis of immunoreactive bands normalized to GAPDH protein levels. ** *p* < 0.01, Student’s *t* test.

As observed for B16-LS9 cells, NSC12 treatment inhibits the phosphorylation of the FGF receptors FGFR1 and FGFR3 as well as of their downstream signaling molecules FRS2 and ERK_1,2_ in the UM cell lines tested (Figure 5A). This resulted in a significant inhibition of UM cell proliferation/survival with an IC_50_ ranging between 6.0 and 8.0 µM NSC12 (Figure 5B). Similar results were obtained after treatment with the selective tyrosine kinase FGFR inhibitor BGJ398 [34] (data not shown).

In keeping with the hypothesis that the FGF system may play a pivotal role in UM cell survival [9], NSC12 induced pro-apoptotic caspase-3 activation and PARP cleavage in UM cells (Figure 5C) that were followed by a significant increase of annexin-V^+^ apoptotic cells (Figure 5D).

β-catenin signaling has been involved in UM cell migration and metastasis [35,36,37]. Notably, NSC12 treatment caused a significant and rapid decrease of the protein levels of β-catenin in UM cells. (Figure 5E). Accordingly, FGF trapping by NSC12 inhibited cell migration in a Boyden chamber chemotaxis assay and following the mechanical wound of a UM cell monolayer (Figure 5F,G). It must be pointed out that both assays were performed under experimental conditions that did not exert a significant effect on UM cell survival (data not shown).

Together, these findings demonstrate that FGF trapping exerts a significant impact on the autocrine FGF/FGFR axis in human UM cells.

## 3. Discussion

In the present work, we demonstrate that different FGF trapping approaches inhibit the tumorigenic and metastatic activity of hepatotropic murine B16-LS9 cells and hamper the proliferation, survival, and migration of primary and metastatic human UM cell lines.

B16-LS9 cells are a murine cell line originated from a B16-F1 liver metastasis and characterized by a unique tropism for the hepatic tissue that recapitulates the metastatic growth patterns observed in the human disease [25]. Indeed, despite its cutaneous origin, B16-LS9 cells have been selectively developed after serial passages for liver specific metastasis, leading to the only model metastasizing to the liver following intraocular injection in syngeneic animals [38]. Thus, B16-LS9 cells have been utilized as an experimental model to investigate the mechanisms responsible for UM liver tropism [26,27,28], drug testing for UM therapy [29,30,31], immunologic and angiogenic aspects of UM and imaging methodologies (see [38] and references therein).

Here, we show that B16-LS9 cells express different FGFRs and the prototypic FGFR ligand FGF2. This leads to the constitutive phosphorylation of FGFR1 and FGFR3, as well as of the downstream signaling proteins FRS2, ERK_1/2_, and AKT. As observed for different FGF-dependent tumor cell types, this activates an autocrine loop of stimulation in B16-LS9 cells, which is inhibited by the overexpression of the natural extracellular FGF trap PTX3 [16,17,21,39]. The capacity of PTX3 to suppress the proliferative and migratory activity of B16-LS9 transfectants highlights the non-redundant role of the autocrine FGF/FGFR system in the tumorigenic activity of these cells [12]. Indeed, PTX3-overexpressing B16-LS9 tumor grafts showed a reduced rate of growth in syngeneic mice and a reduced metastatic activity when injected in the blood stream of zebrafish embryos.

To assess the effect of the systemic delivery and stromal accumulation of PTX3 protein on UM growth, we took advantage of TgN (Tie2-hPTX3) mice, a transgenic mouse line we generated in the C57BL/6 background that expresses PTX3 under the control of the endothelial specific *Tie2/Tek* transcription regulatory sequences [21]. When injected s.c. in TgN (Tie2-hPTX3) mice, B16-LS9 cells showed a reduced rate of growth, thus confirming the oncosuppressive effect exerted by PTX3 on these cells. Notably, the systemic accumulation of PTX3 protein resulted also in a significant inhibition of the capacity of B16-LS9 cells to originate liver metastases when injected into the spleen of the transgenic animals. Even though we cannot rule out the possibility that PTX3 may have multiple impacts on tumor growth, the data support the notion that the anti-tumor effects of PTX3 are related to its inhibitory action on the autocrine/paracrine loops of stimulation triggered by the FGF/FGFR system in FGF-dependent B16-LS9 cells.

Despite its oncosuppressive effects, the complex proteinaceous structure of PTX3 hampers its pharmacological exploitation. For this reason, the scaling down of this macromolecule to PTX3-derived small molecules was attempted to take advantage of its antitumor properties in a translational outlook. This led to the identification of the small molecule NSC12 as the first orally available pan-FGF trap endowed with a potent anti-tumor activity in different FGF-dependent tumor models (reviewed in [7]). On this basis, a series of experiments were performed to assess the effect of NSC12 on B16-LS9 cells. In keeping with its FGF trapping activity, NSC12 inhibits FGFR phosphorylation, proliferation, and migration of B16-LS9 cells. Notably, NSC12 was able to suppress the growth of these cells also when orthotopically implanted in the eye of zebrafish embryos or when injected into the liver of syngeneic mice.

Even though the syngeneic B16-LS9 model shows significant experimental advantages and resemblance to UM behavior concerning its hepatotropic features, significant genetic differences occur between cutaneous and UM [40,41]. This prompted us to assess the impact of the FGF trapping activity of NSC12 on both primary and metastatic human UM cell lines. Notably, NSC12 treatment was able to inhibit FGFR activation and downstream signaling in all the cell lines tested. This was paralleled by the activation of the pro-apoptotic proteins PARP and caspase-3, thus leading to UM cell death.

The β-catenin signaling pathway has been involved in the growth, migratory, and invasive behavior of UM cells [35]. Indeed, β-catenin immunoreactivity is increased in primary UM and is associated with a shorter patient survival [36], its expression representing a biomarker potently correlated to metastatic UM [37]. The FGF/FGFR axis has been shown to stabilize β-catenin, leading to its nuclear accumulation and activation of the β-catenin signaling pathway [42,43]. Notably, our data demonstrate that NSC12 induces a decrease of β-catenin levels in UM cells that was paralleled by a significant inhibition of UM cell migration in a Boyden chamber assay or following the mechanical wound of the cell monolayer. Further studies will be required to dissect the impact of β-catenin downregulation induced by FGF inhibitors on the tumorigenic and metastatic behavior of UM cells.

In keeping with our preclinical observations, the analysis of the publicly available mRNA profiling dataset of 80 primary human UM specimens present in TCGA indicates that the upregulation of different members of the FGF or FGFR families are associated with poorer prognosis, chromosome 3 monosomy, and *BAP1* mutation. These data further support the hypothesis that the FGF/FGFR system plays a non-redundant role in UM. Several experimental evidences reinforce this assumption. Similar to NSC12, neutralizing antibodies and an antisense oligonucleotide directed against FGF2 have been shown to reduce cell proliferation and survival in various human UM cell lines [12]. When examined on an array of 32 human UM samples, FGF2 immunoreactivity was detectable in more than 50% of cases, its frequency being higher in mixed/epithelioid samples than in spindle cell type specimens [13]. Notably, immunohistochemical staining revealed an elevated FGF2 expression in UM metastases when compared to primary lesions, further implying FGF2 in UM progression [13]. In addition, the production of FGF2 by UM cells and primary tumors may contribute, together with vascular endothelial growth factor, to the angiogenic activity of UM that, in turn, favors its hematogenous metastatic spread [10,11]. Finally, recent observations have shown that FGF2 produced by hepatic stellate cells confers resistance of metastatic UM cells to bromodomain and extraterminal protein inhibitors and to the histone deacetylase inhibitor vorinostat via FGFR activation [44]. Together with our observations, these data suggest that drugs targeting the FGF/FGFR system might be considered for an adjuvant chemotherapy treatment of metastatic UM.

Thus far, different approaches have been developed to target the FGF/FGFR system [9] and various FGF/FGFR inhibitors are under evaluation in clinical trials on cancer patients affected by different kinds of tumors [8]. In this frame, drugs targeting FGF ligands may represent an interesting alternative to tyrosine kinase FGFR inhibitors. To this respect, the small molecule NSC12 is the first orally active multi-FGF trap. Of note, in keeping with the lack of pathological consequences following constitutive PTX3 overexpression in transgenic mice, the anti-tumor action of NSC12 occurs in the absence of any significant effect on body weight and survival of treated animals [21]. Thus, NSC12 may represent a lead compound for the development of orally active small molecule therapeutics for the treatment of UM in which the ligand-dependent activation of the FGFR pathway is an oncogenic driver. Further experiments aimed to assess the in vivo efficacy of this FGF trapping approach in orthotopic and liver metastatic models of human UM are required to confirm this hypothesis.

## 4. Materials and Methods

### 4.1. Reagents

All reagents were of analytical grade. Dulbecco’s modified Eagle medium (DMEM), RPMI 1640 medium and fetal bovine serum (FBS) were from GIBCO Life Technologies (Grand Island, NY, USA). Penicillin, streptomycin, Triton-X100, BriJ, sodium orthovanadate, protease inhibitor cocktail, and anti-α-tubulin were from Sigma-Aldrich (St. Louis, MO, USA). Bradford reagent was from Bio-Rad Laboratories (Milan, Italy). Trizol, MMLV reverse transcriptase and CellTracker Red CMTPX Dye were from Invitrogen (Carlsbad, CA, USA). PVP-free polycarbonate filters were obtained from Costar (Cambridge, MA, USA). Diff-Quik reagent was obtained from Dade-Behring (Deer eld, IL, USA). ONE-Glo™ Reagent and DNAse were from Promega (Milan, Italy). Recombinant FGF2 was purchased from Tecnogen (Caserta, Italy). Anti-FGFR1, anti-ERK_1/2_, anti-phospho-ERK_1/2_ (Thr202/Tyr204), anti-phospho-AKT (Ser473), anti-cleaved-PARP, anti-cleaved-caspase-3, and anti-β-catenin were from Cell Signaling Technologies (Danver, MA, USA). Anti-phospho-FGFR1 (Tyr766), anti-phospho-FRS2 (Tyr196), anti-FGFR3, and anti-GAPDH were from Santa Cruz (Santa Cruz, CA, USA). Anti-phospho-FGFR3 (Tyr724) was from ABCAM (Cambridge, UK). Matrigel was from Cultrex BME (Gaithersburd, MD, USA). Floseal hemostatic matrix was from Baxter (Deerfield, IL, USA).

### 4.2. Cell Cultures

Murine B16-LS9 cells [25] were maintained in DMEM supplemented with 10% FBS, 100 U/mL penicillin, and 100 μg/mL streptomycin. B16-LS9 cells were transfected with a pBABE/Puro vector harboring the full-length human PTX3 cDNA or with empty vector as described [19]. Stable hPTX3_LS9 and mock_LS9 cell populations were generated by puromycin selection. Luciferase-transfected B16-LS9 cells (B16-LS9-*luc* cells) were generated as described [21]. Human UM cells [45,46,47] were maintained in RPMI 1640 medium supplemented with 10% (Mel285, 92.1, and OMM2.3 cells) or 20% FBS (Mel270 cells), 100 U/mL penicillin, and 100 μg/mL streptomycin.

### 4.3. Real-Time PCR Analysis

For mRNA expression analysis, cells were processed, and total RNA was extracted using TRIzol Reagent according to manufacturer’s instructions. Contaminating DNA was digested using DNAse and 2.0 μg of total RNA were retro-transcribed with MMLV reverse transcriptase using random hexaprimers in a final 20 μL volume. Then, 1/10th of the reaction was analyzed by semiquantitative RT-PCR using specific primers (Table 2). The PCR products were then electrophoresed on a 1.5% agarose gel and visualized by ethidium bromide staining.

### 4.4. Western Blot Analysis

For the analysis of FGF signaling, cell samples were homogenized in RIPA buffer containing 1.0% Triton-X100, 0.2% BriJ, 1.0 mM sodium orthovanadate, and protease inhibitor cocktail. Protein concentrations were determined using the Bradford protein assay. Western blot analysis was performed using rabbit anti-FGFR1, anti-pFGFR1, anti-FGFR3, anti-pFGFR3, anti-pFRS2, anti-pAKT, anti-ERK_1/2_, anti-pERK_1/2_, anti-cleaved-PARP, anti-cleaved-caspase-3, anti-β-catenin antibodies, and normalized with anti-α-tubulin or anti-GAPDH antibodies. Densitometric analysis was performed using the Bio-Rad Image Lab Software 5.2.1. The whole blot showing all the bands with all molecular weight markers on the Western are included in the Supplementary Materials.

### 4.5. Cell Proliferation Assay

Cells were seeded on 48-well plates at 1 × 10^4^ cells/cm^2^ (B16-LS9 cells) or at 1.5 × 10^4^ cells/cm^2^ (Mel285, Mel270, 92.1, and OMM2.3 cells). After 24 h, cells were treated with increasing concentrations of NSC12. After a further 24 or 48 h incubation, cells were trypsinized and viable cell counting was performed with the MACSQuant^®^ Analyzer (Miltenyi Biotec) as reported [48].

### 4.6. Colony Formation Assay

B16-LS9 cells were seeded in 35-mm culture dishes at 50 cells/cm^2^. After 10 days of incubation, cell colonies were stained with crystal violet and quantified by computerized image analysis.

### 4.7. Chemotaxis Assay 

Mel285 cells were seeded at 1.0 × 10^6^ cells/mL in the upper compartment of a Boyden chamber containing gelatin-coated PVP-free polycarbonate filters (8 μm pore size). RPMI medium supplemented with 1.0% FBS was placed in the lower compartment in the absence or in the presence of 6.0 µM NSC12. After 4 h of incubation at 37 °C, cells that migrated to the lower side of the filter were stained with Di-Quik reagent. Five random fields were counted for each triplicate sample.

### 4.8. Wound Healing Assay

Confluent B16-LS9 or Mel285 cells were scraped with a 200 μL tip to obtain a mechanical wound through the cell monolayer. Then, B16-LS9 cells were maintained in DMEM supplemented with 0.4% FBS in the absence or in the presence of 3.0 µM NSC12 whereas Mel285 cells were maintained in RPMI medium supplemented with 1.0% FBS and treated or not with 6.0 µM NSC12. After 18 h, cells at the leading edge of the wound were photographed under an inverted Zeiss Axiovert 200 M photomicroscope and cell migration wound was quantified by computerized image analysis.

### 4.9. Apoptotic Cell Death Analysis

Mel285 or 92.1 cells were seeded at 2.5 × 10^5^ cells/mL in the absence or in the presence of 15 µM NSC12. After 12 h, apoptotic cell death was assessed by cytofluorimetric analysis following Annexin-V/propidium iodide-double staining according to manufacturer’s instructions.

### 4.10. Tumor Graft and Liver Metastasis Assays in Mice

Animal studies were approved by the local animal ethics committee (OPBA, Organismo Preposto al Benessere degli Animali, Università degli Studi di Brescia, Italy) and by the Italian Ministero della Salute (Project: Integrated model for the study and therapy of uveal melanoma; authorization no. 1306/2015-PR). All the procedures and animal care were conformed to institutional guidelines that comply with national and international laws and policies (EEC Council Directive 86/609, OJ L 358, 12 December 1987).

C57BL/6 (Charles River, Calco, Italy) and transgenic TgN (Tie2-hPTX3) mice [21] were maintained under standard housing conditions.

B16-LS9 cells were injected s.c. or into the spleen [28] of wild-type and TgN (Tie2-hPTX3) mice at 50,000 and 20,000 cells/graft, respectively. Subcutaneous tumors were measured with calipers and tumor volume was calculated according to the formula V = (D × d2)/2, where D and d are the major and minor perpendicular tumor diameters, respectively. Liver metastases were counted under a dissecting microscope 14 days after cell injection.

As for liver tumor grafts, 20 µL of a cell suspension containing 50,000 B16-LS9-*luc* cells in Matrigel (1:1, vol/vol) were injected into the liver of wild-type mice. To minimize bleeding and to avoid leakage of tumor cells after needle removal, Floseal hemostatic matrix was applied on the liver surface at the site of injection. NSC12 treatment (7.5 mg/kg) was performed every other day by i.p. injection in a 100 μL final volume. Tumor growth was imaged with IVIS Lumina III during the following 14 days.

### 4.11. Zebrafish Embryo Assays

The AB zebrafish line was maintained at 28 °C on a 14 h light/10 h dark cycle. After spawning, fertilized eggs were harvested and incubated in fish water at 28 °C. Zebrafish embryos were staged and maintained in 0.003% 1-phenyl-2-thiourea (Sigma) starting from 24 hpf to prevent pigmentation.

For the metastasis assay, B16-F10 cells were stained with the red fluorescent CellTracker Red CMTPX Dye. Then, 80–100 cells/embryo were injected into the blood circulation in the ventral region of the duct of Cuvier of zebrafish embryos at 48 hpf. During the next 3 days, the growth of fluorescent metastases in the tail vascular plexus was quantified by fluorescence microscopy followed by computerized image analysis of the fluorescent tumor area [32].

For the orthotopic UM model, B16-LS9-*luc* cells (100 cells/embryo) were injected into the eye of zebrafish embryos at 48 hpf using a borosilicate needle and an Eppendorf FemtoJet microinjetor equipped with an InjectMan NI2 manipulator. Then, embryos were transferred in fish water in the absence or in the presence of increasing concentrations of NSC12. Tumor growth was evaluated 3 days after grafting by measuring the cell luminescence signal as it follows: embryos were anesthetized and singularly placed in a well of a white polystyrene 96-well plate; medium was removed and replaced with 50 µL of RIPA buffer plus 50 µL of ONE-Glo™ Reagent; finally, luminescence was measured using an EnSight multimode plate reader (Perkin Elmer, Waltham, MA, USA).

### 4.12. Statistical Analysis

Statistical analysis was performed with GraphPad Prism 7 (San Diego, CA, USA) using Student’s *t*-test or one-way analysis of variance followed by Bonferroni multiple comparison post-test. Tumor growth data were analyzed by two-way analysis of variance, followed by Bonferroni post-test. Differences were considered significant when *p* values < 0.05.

## 5. Conclusions

We demonstrate that the natural extracellular FGF trap PTX3 inhibits the oncogenic activity of hepatotropic murine melanoma B16-LS9 cells. Translational exploitation of these findings shows that the PTX3-derived pan-FGF trap small molecule NSC12 hampers the tumorigenic and liver metastatic activity of B16-LS9 cells and affects autocrine FGF/FGFR signaling, proliferation, survival, and migration of human UM cells. Together, these findings indicate that FGF trapping may represent a novel therapeutic strategy for the treatment of metastatic UM.

## Figures and Tables

**Table 1:** Molecular alterations of the human UM cell lines utilized in this study.

	Mel285	92.1	Mel270	OMM2.3
*GNAQ* (exons 4–5)	WT	Q209L (626 A > T)	Q209P (626 A > C)	Q209P (626 A > C)
*GNA11* (exons 4–5)	Q209L	WT	WT	WT
*BAP1*	WT	WT	WT	WT
BAP1	Yes, low	Yes	Yes	Yes
*SF3B1*	WT	WT	WT	WT
*EIF1AX*	WT	c.17G/A	WT	WT
Chr3	Disomy 3 Loss 3p26-pter	Disomy 3	Disomy 3 Loss 3p24 Loss 3q21.2-3q24	Disomy 3 Loss 3p24 Loss 3q21.2-3q24
Chr6	Disomy 6p	Gain 6p	Tetrasomy 6p	Tetrasomy 6p
Chr8	Disomy 8p Tetrasomy 8q	Gain 8q	Disomy 8 Extra 8q	Disomy 8 Extra 8q

**Table 2:** Oligonucleotide primers used for RT-PCR analysis.

Gene	Forward	Reverse
*Fgf2*	5’-CCTTCCCACCAGGCCACTTAA-3’	5’-GGTCCGTTTTGGATCCGAGTTT-3’
*Fgfr1*	5’-GCTGACTCTGGCCTCTACGCT-3’	5’-CAGGATCTGGACATACGGCAA-3’
*Fgfr2*	5’-CTGCCTGGTGGAGAATGAAT-3’	5’-CGCTGTAAACCTTGCAGACA-3’
*Ffgr3*	5’-CTGAAGCACGTGGAAGTGAA-3’	5’-CCTCAAAGGTGACATTGTGC-3’
*Fgfr4*	5’-ACTGTCAAATTCCGCTGTCC-3’	5’-AGCGAATGCTACCCAGAGAG-3’
*PTX3*	5’-CATCTCCTTGCGATTCTGTTTTG-3’	5’-CCCATTCCGAGTGCTCCTGA-3’
*Gapdh*	5’-GAAGGTCGGTGTGAACGGATT-3’	5’-TGACTGTGCCGTTGAATTTG-3’

## Supplementary Materials

The following are available online at https://www.mdpi.com/2072-6694/11/9/1305/s1, The whole blot showing all the bands with all molecular weight markers on the Western.
