# Supplementary material for: The Autocrine FGF/FGFR System in both Skin and Uveal Melanoma: FGF Trapping as a Possible Therapeutic Approach
Source: Cancers (Basel). 2019 Sep 4;11(9):1305. doi: 10.3390/cancers11091305 (PMC6770058; doi:10.3390/cancers11091305)
Supplement: Supplementary file 1 [file cancers-11-01305-s001.pdf]

Figure 2B

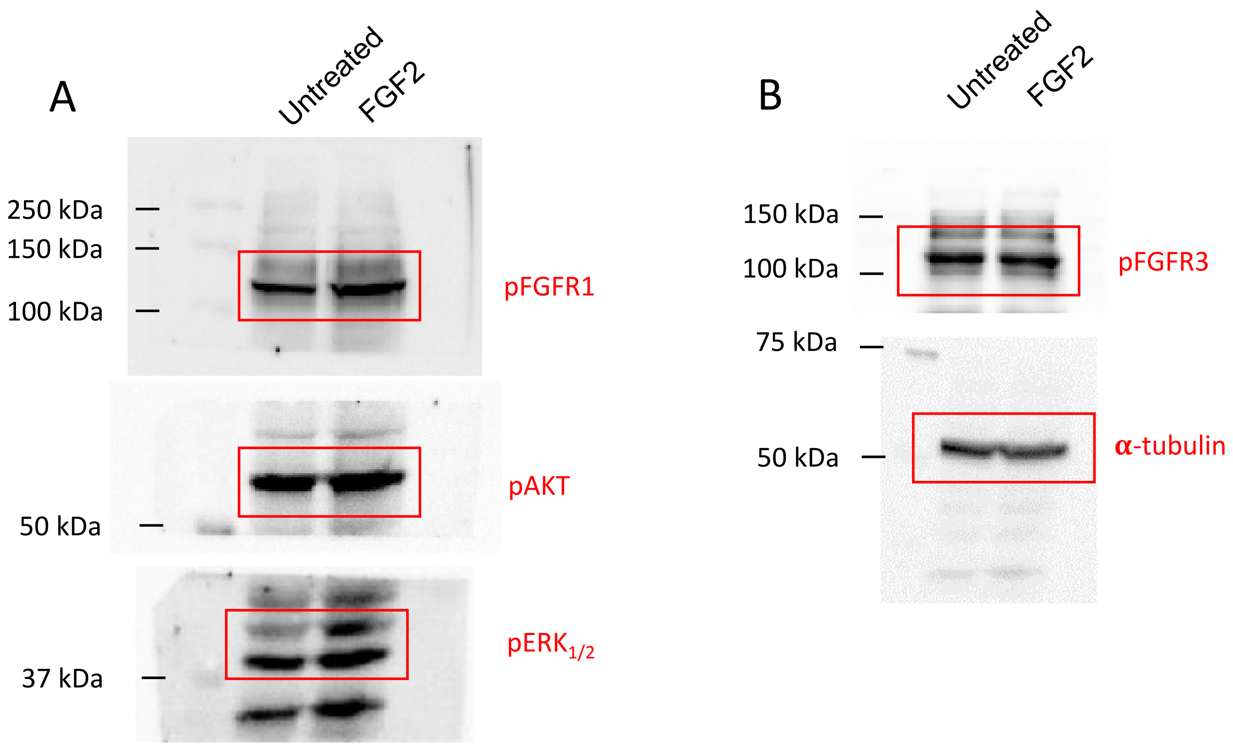

A) After transfer the membrane was cut and incubated with the indicated Abs and acquired separately with a BioRad ChemiDoc Imaging System. **B)** Then, a second aliquot of the same cell extracts was loaded on a different gel and incubated with the indicated Abs.

Figure 2D

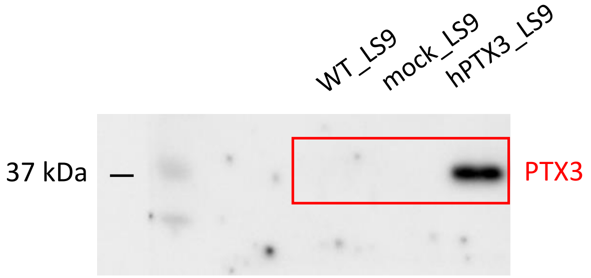

Cell extracts were prepared from the indicated cell lines. After transfer the membrane was cut, incubated with the indicated Ab and acquired with a BioRad ChemiDoc Imaging System.

Figure 2E

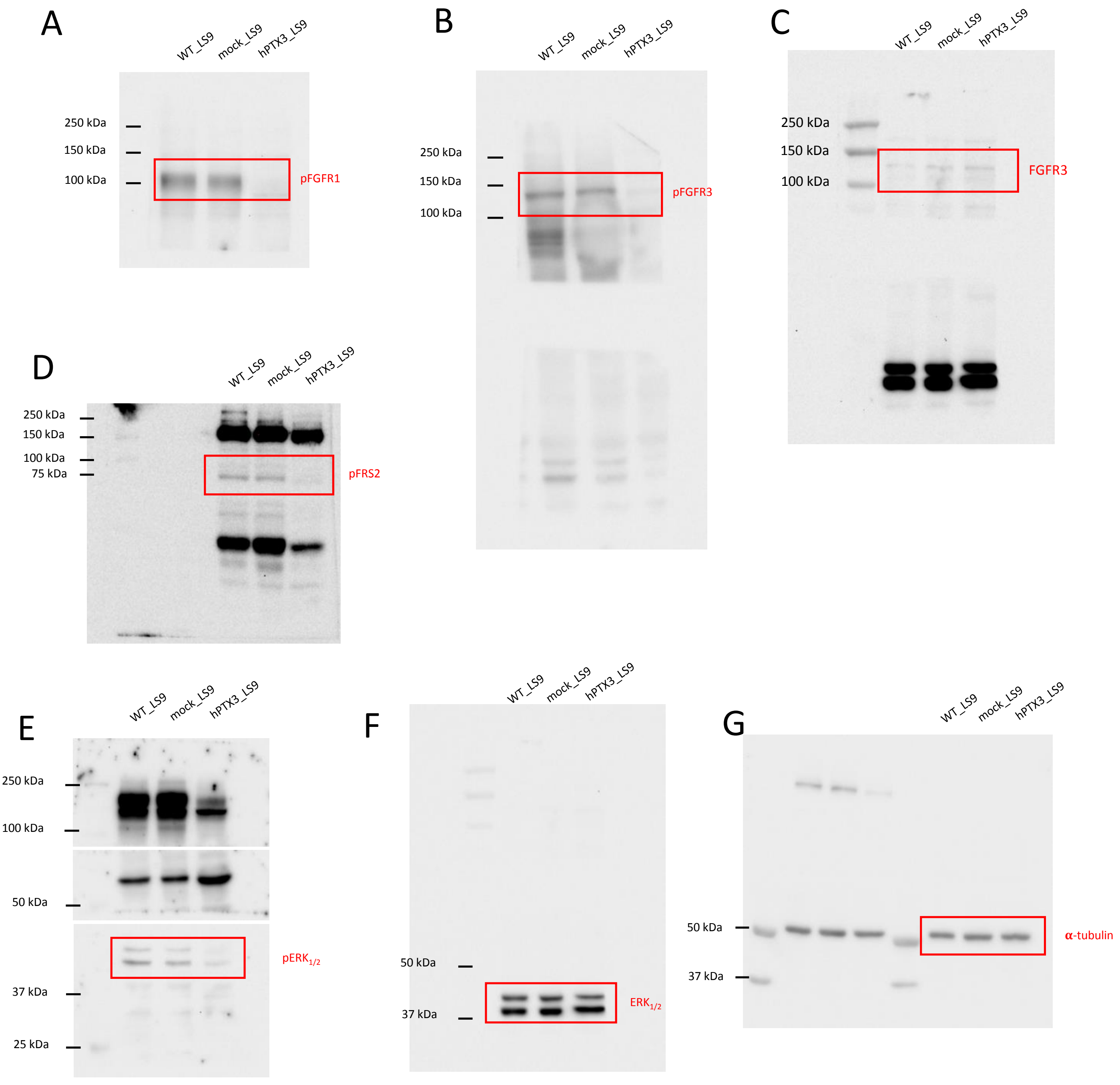

A-G) Cell extracts were prepared from the indicated cell lines. Then, aliquots from the same preparation were loaded on different gels. Each membrane was cut as appropriate and incubated with the indicated Ab. C and F are the same membrane at two different times of acquisition.

Figure 3A

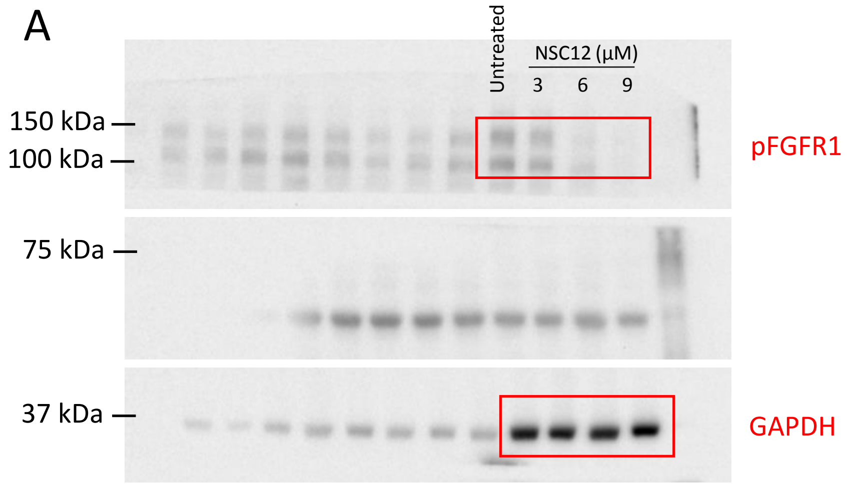

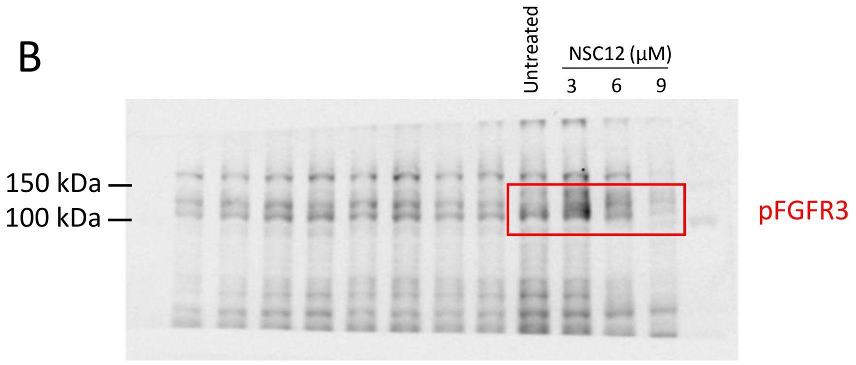

A, B) Cell extracts were prepared following the indicated treatment. Then, aliquots from the same preparation were loaded on the two gels. Each membrane was cut as appropriate and incubated with the indicated Ab.

Figure 4A

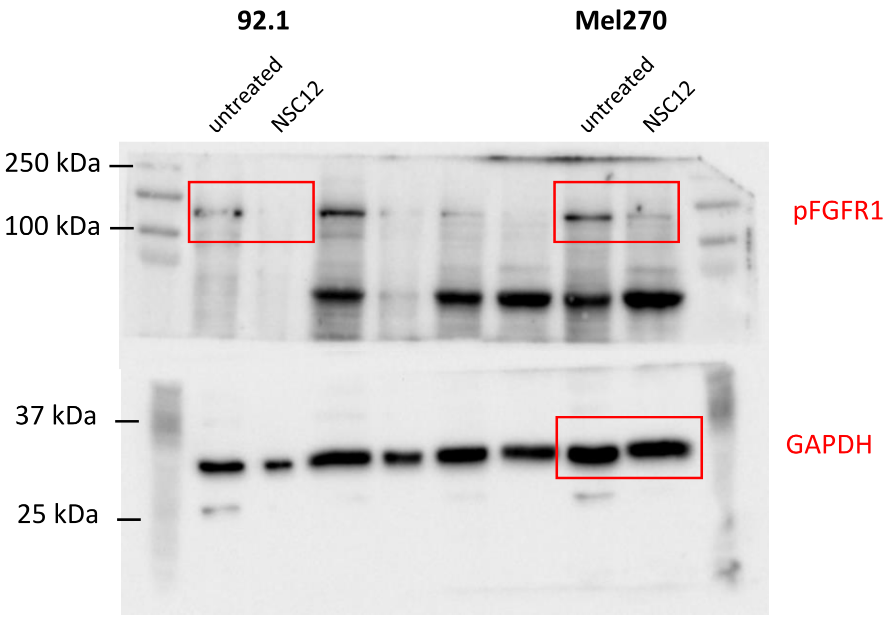

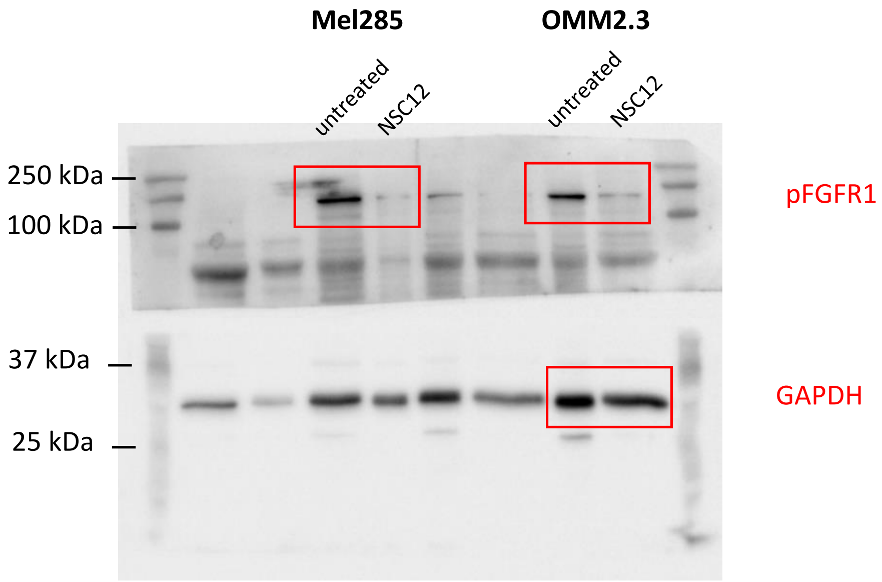

After transfer each membrane was cut, incubated with the indicated Abs and acquired separately with a BioRad ChemiDoc Imaging System.

Figure 4A

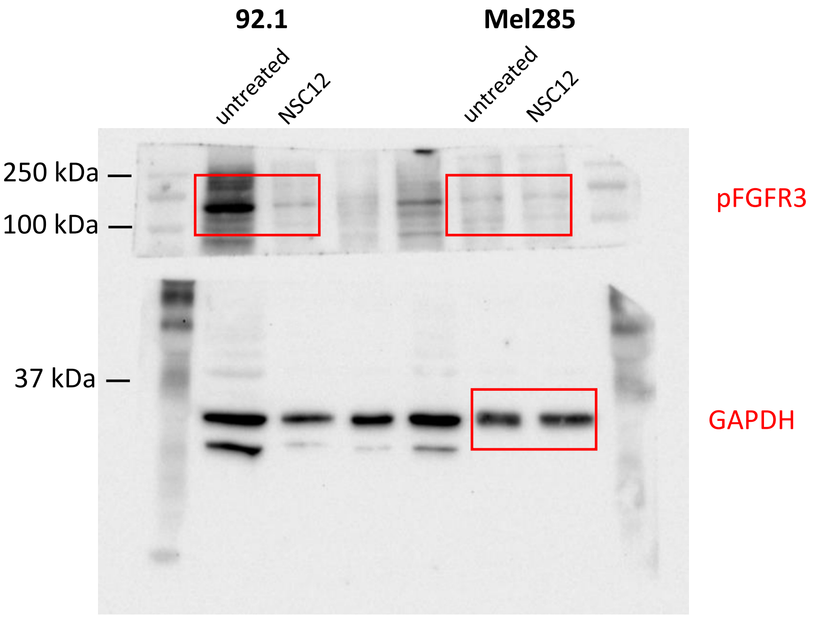

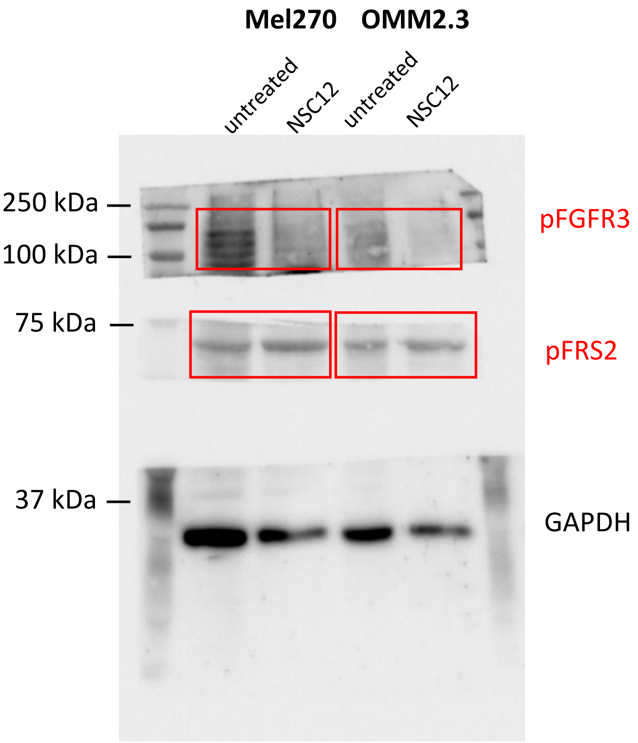

After transfer each membrane was cut, incubated with the indicated Abs and acquired separately with a BioRad ChemiDoc Imaging System.

Figure 4A

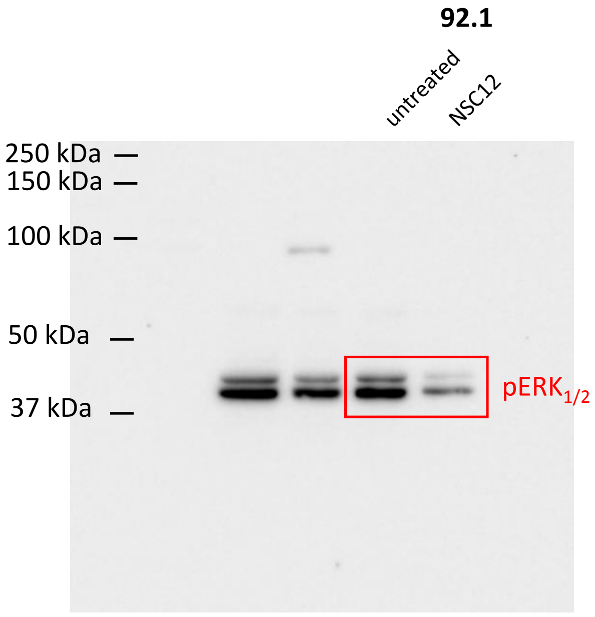

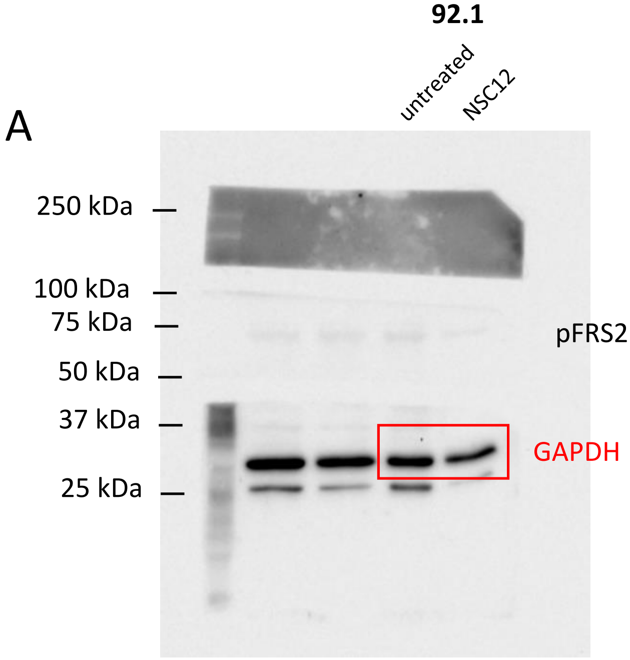

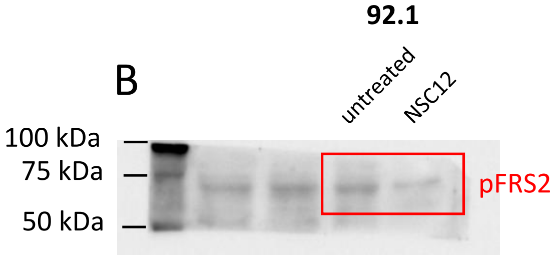

After transfer each membrane was cut, incubated with the indicated Abs and acquired separately with a BioRad ChemiDoc Imaging System. A and B are the same membrane at two different times of acquisition.

Figure 4A

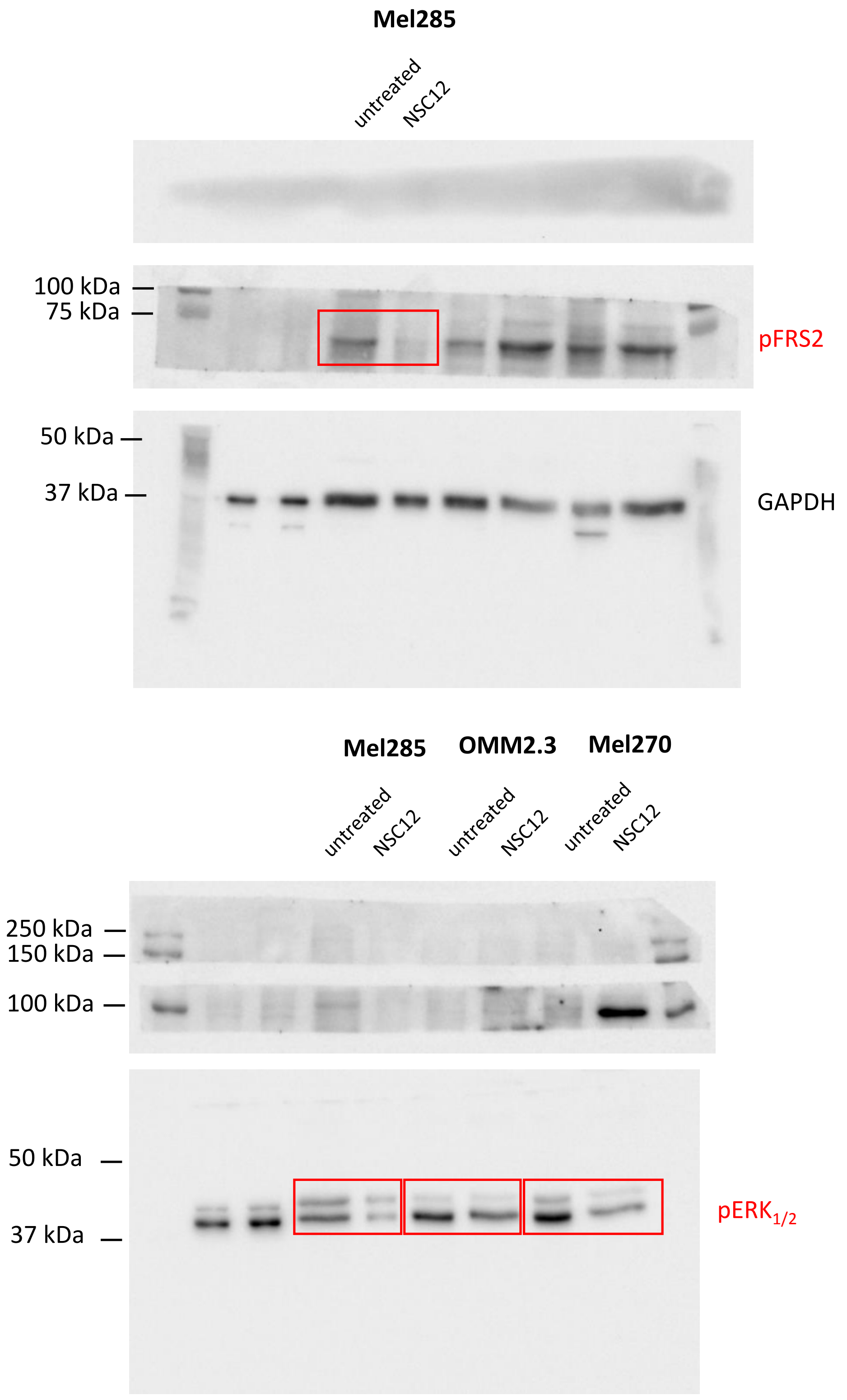

After transfer each membrane was cut, incubated with the indicated Abs and acquired separately with a BioRad ChemiDoc Imaging System.

Figure 4C

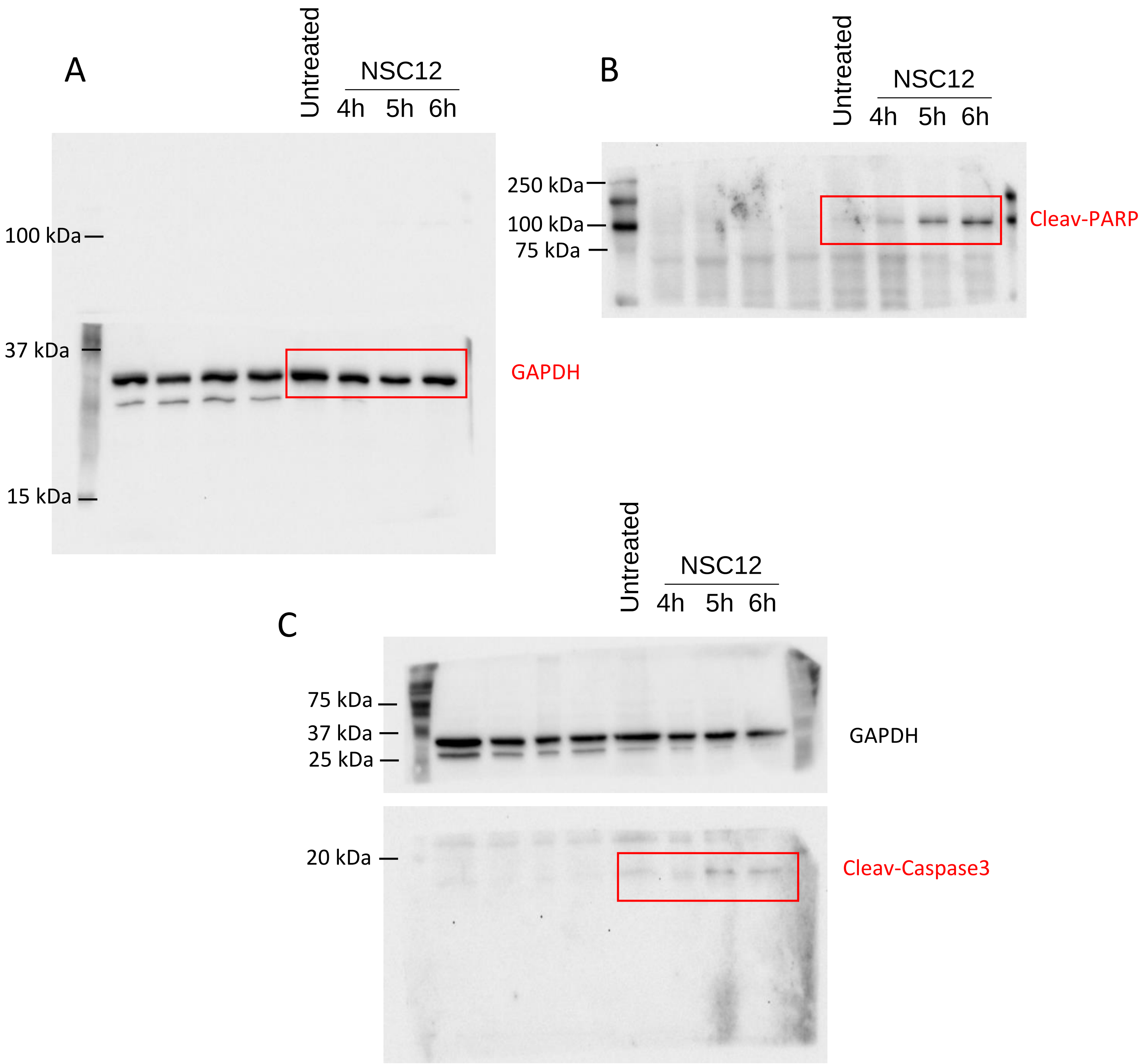

After transfer each membrane was cut, incubated with the indicated Abs and acquired separately with a BioRad ChemiDoc Imaging System. A and B are the same membrane at two different times of acquisition.

Figure 4E

A

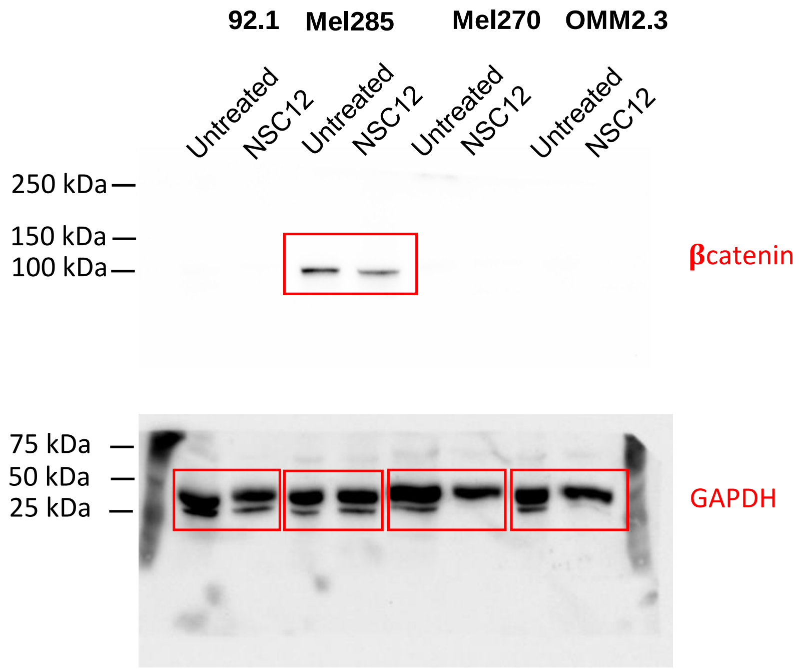

B

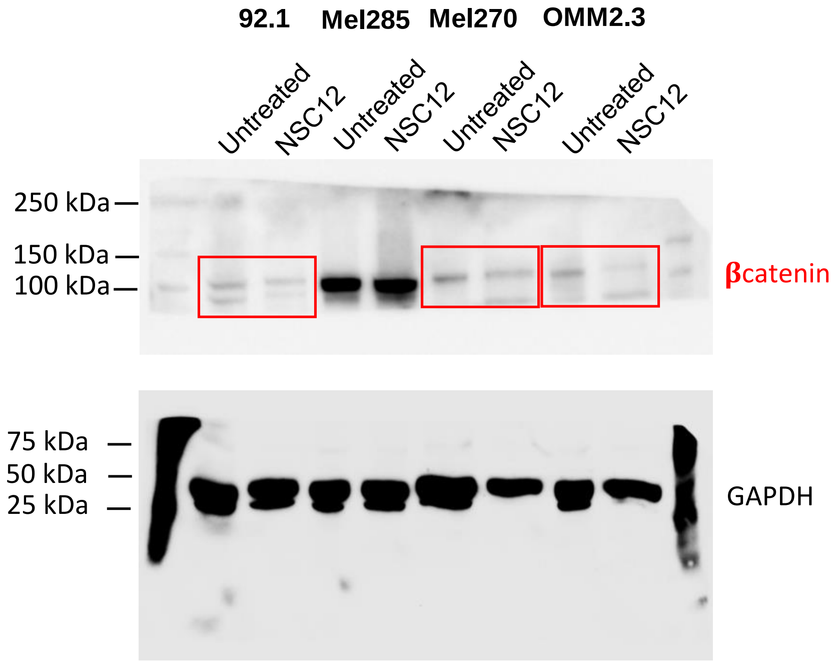

After transfer each membrane was cut, incubated with the indicated Abs and acquired separately with a BioRad ChemiDoc Imaging System. A and B are the same membrane at two different times of acquisition.
